# The epigenome: the next substrate for engineering

**DOI:** 10.1186/s13059-016-1046-5

**Published:** 2016-08-31

**Authors:** Minhee Park, Albert J. Keung, Ahmad S. Khalil

**Affiliations:** 1 Department of Biomedical Engineering and Biological Design Center, Boston University, Boston, MA 02215 USA; 2 Department of Chemical and Biomolecular Engineering, North Carolina State University, Raleigh, NC 27695 USA; 3 Wyss Institute for Biologically Inspired Engineering, Harvard University, Boston, MA 02115 USA

## Abstract

We are entering an era of epigenome engineering. The precision manipulation of chromatin and epigenetic modifications provides new ways to interrogate their influence on genome and cell function and to harness these changes for applications. We review the design and state of epigenome editing tools, highlighting the unique regulatory properties afforded by these systems.

## Introduction

Chromatin is decorated by a large array of biochemical modifications made to DNA and histone proteins [1]. These modifications—and the broader organizational structure of chromatin—provide an important additional layer of information that is superimposed upon genome sequence and thus are widely referred to as the epigenome. Given its physical association with genomic material, the epigenome has been suggested to play key roles in regulating genome structure and function, including the timing, strength, and memory of gene expression [2–4]. The epigenome is thought to help control which genes are expressed in a given context, for example, to produce the gene expression patterns that underlie the many different cellular phenotypes that arise during an organism’s development. Because many modifications are heritably maintained, the epigenome is also believed to be key in determining how these gene expression patterns are subsequently maintained for the life of an organism. Moreover, a large body of evidence suggests that the epigenome is inappropriately altered in many human diseases, including most cancers [5–8].

Yet, there remains much that we do not understand about the function of the epigenome. Recently, with the advent of genomic techniques, there has been remarkable progress in our ability to map epigenomic modifications at a global scale and to correlate them with gene expression. While the roles of many chromatin modifications remain unclear, some important patterns have begun to emerge in which epigenome states have come to define key signatures of gene regulation, cell activity, and even disease states [2, 3]. Despite these significant advances, many questions remain unresolved, especially concerning the cause and consequence of chromatin marks with respect to gene expression and other regulatory processes. Thus, the stage is set for the development of new methods that can selectively manipulate and probe the epigenome. Tools that can be used to edit chromatin modifications at specific locations and times will deepen our functional understanding of the epigenome, for example, by allowing researchers to directly interrogate the relationship between the epigenome and transcriptional control. They will also provide opportunities to transform the increasingly precise genome-wide maps that have been generated for developmental and disease states into therapeutics and other benefits for human health.

**Fig. 1:**
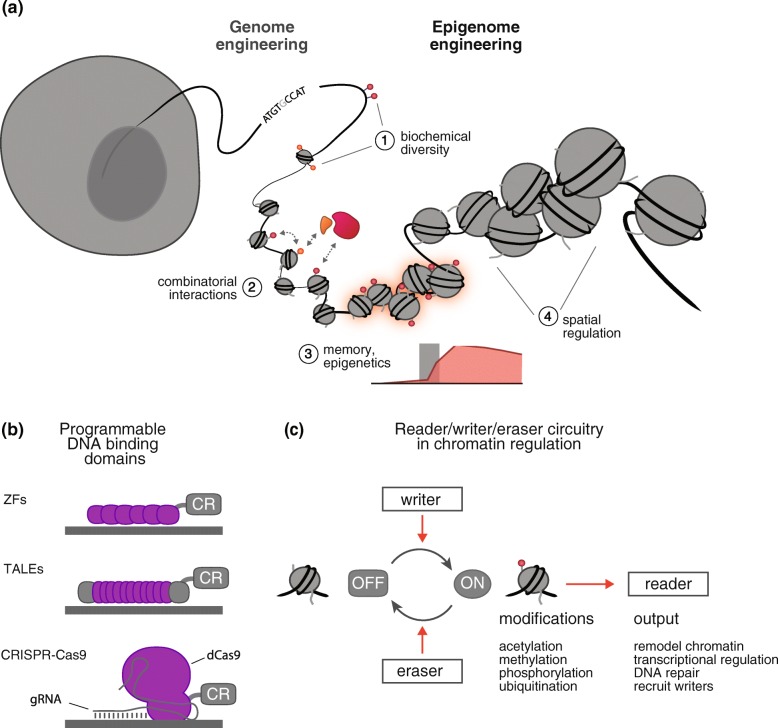
Epigenome engineering is the selective manipulation of chromatin and epigenetic modifications in the genome. **a** Epigenetic modifications provide a rich set of capabilities and challenges for engineering, including 1) a large biochemical diversity, 2) a preponderance of combinatorial interactions, 3) the potential for long-term memory, and 4) the ability to regulate genes over large spatial ranges. **b** Programmable DNA-binding domains, which have been used extensively in genome engineering applications and are now being harnessed to design epigenome engineering tools. Epigenetic editors are fusions of a DNA-binding module (zinc fingers (*ZFs*), transcription activator-like effectors (*TALEs*) or CRISPR-Cas9) to one or more chromatin regulator (*CR*) modules. Each ZF domain recognizes ~3–4 nucleotide sequences, whereas each TALE domain recognizes a single nucleotide. The Cas9 protein is directed to its target site by an engineered guide RNA (*gRNA*) that binds genomic sequences via Watson–Crick base pairing. *dCas9* nuclease-null Cas9 protein. **c** The manipulation of chromatin and epigenetic modifications can be understood in terms of reader/writer/eraser schemes. Molecular writers and erasers serve to catalyze the transfer and removal of chemical marks on target histone residues. The mark is then interpreted by readers, which function to recruit and/or alter functionality. Inspired by and adapted from [9]

At the center of these new efforts are the programmable DNA-targeting technologies behind the genome engineering revolution: zinc fingers (ZFs), transcription activator-like effectors (TALEs), and the CRISPR/Cas systems. These technologies are now being utilized for targeted epigenome editing through the recruitment of functional domains to DNA sequences of interest (Fig. 1). Chromatin is, however, an incredibly complex and dynamic regulatory system, which offers both unique opportunities and challenges for this class of technologies. Here, we review the current state of epigenome engineering. Specifically, we discuss new tools and approaches that have allowed researchers to address, interrogate, and reprogram four key features of chromatin: (1) the biochemical diversity of chromatin modifications, (2) the combinatorial and context-dependent nature of chromatin modifications, (3) the memory and long-term stability of modifications, and (4) the potential for long-range spatial regulation (Fig. 1). Throughout, we highlight key design considerations and challenges and suggest strategies for addressing them. We pose ways in which these functional tools can be expanded to help to answer fundamental questions about gene and cellular regulation and we tackle a range of application spaces. Finally, we note that synthetic control over chromatin provides new capabilities in the field of synthetic biology, the engineering of functional biological systems from genetically encoded “parts”. New possibilities include engineering higher-order transcriptional control in cells and programming cellular memory states through the manipulation of epigenetic marks. The development of engineered readers, writers, and erasers that can effectively process the reversible modifications made to chromatin will expand the synthetic biology toolkit available for establishing synthetic linkages in cellular networks, enabling a better understanding of the function of these networks and control of complex cellular behaviors (Fig. 1) [9, 10].

## Biochemical diversity: selecting modifications and substrates

To explore and exploit the functional roles of DNA and histone modifications, new tools are being developed to selectively alter chromatin biochemistry at specific genomic loci. One striking feature of chromatin is the large biochemical diversity in the modifications and their substrates [4, 11]. For example, with histone modifications, a variety of residues displayed on histone tails act as substrates for a range of post-translational modifications (PTMs), including methylation, acetylation, phosphorylation, and ubiquitination. A leading hypothesis to explain this biochemical diversity is that the marks (individual and/or in combination) comprise a code that is read by modular reader domains in order to drive specific transcriptional and remodeling functions [12]. This form of regulation has the potential for vast combinatorial power. From the standpoint of designing epigenome editors, this diversity requires that the biochemical specificities (both the type of chemical modification and the target residue) are defined carefully. The location within the genome at which these modifications are made is another important consideration, because different genomic loci exhibit distinct chromatin modifications depending on developmental and cell states. Thus, another key factor in the design of editors is genome site or locus specificity.

### Rapid advances in targeted epigenome editors

Cells use a system of chromatin effectors and associated histone and DNA modifications to modulate and establish gene-expression states. A central goal has been to try to link these modifications to specific functional roles, such as transcriptional activation and repression [2, 3, 13]. To date, our knowledge of chromatin-effector functions has largely derived from the pharmacological inhibition or genetic knockout of histone-modifying enzymes. More recently, precise and comprehensive genome-wide maps of chromatin modifications have been generated, mapped to transcriptomes, and used to provide further correlative evidence for chromatin functions [14]. Nevertheless, these two approaches—genome-wide perturbations and mapping analyses—neither account for potential pleiotropic effects nor directly demonstrate causal relationships between chromatin and functional states. Therefore, in order to complement these studies and to acquire causal and functional connections between chromatin modifications and their putative functions systematically, we need approaches that can selectively perturb chromatin biochemistry at specific genomic loci.

The advent of programmable DNA-targeting technologies, including ZFs [15], TALEs [16–18], and the CRISPR/Cas systems [19–21], has begun to make this possible. These technologies have been used, with tremendous success and excitement, to create programmable nucleases for genome editing in a wide range of cells and organisms [15, 16, 22–24]. The ability to target specific DNA sequences in eukaryotic genomes is now being harnessed to explore whether the epigenome can be similarly edited in a site-specific manner. The basic design of an epigenome editor is a fusion of a DNA-targeting module to one or more chromatin regulators (CRs; Fig. 1b). To date, efforts have largely focused on creating programmable writers (fusions to enzymes that catalyze chemical modifications of DNA or histone residue(s)) and erasers (fusions to enzymes that remove chemical modifications) (Table 1).

**Table 1:** Molecular writers and erasers of chromatin modifications

Modification	Substrate specificity	Putative functions	Example proteins/domains	Epigenetic engineering applications			
				Target locus	DBD	CR fusion	Reference
**DNA**							
Methylation							
Writer	Cytosine	Repression	DNMT1, DNMT3	Human endogenous promoter	ZF	hDnmt3a CD	[25]
				Reporter plasmid in mammalian cells	Gal4, ZF	mDnmt3a CD, mDnmt3b CD	[26]
				Mutated mitochondrial DNA	ZF	hDNMT3a CD	[27]
Eraser	Cytosine	Activation	TET1, TET2, TDG	Human endogenous promoter	ZF	TET1 CD, TET2 CD	[28]
				Human endogenous promoter	TALE, ZF	TET1 FL, CD	[29]
				Mouse endogenous promoter	RHD	TDG FL	[30]
				Mouse endogenous promoter	ZF	TDG FL	[31]
**Histones**							
Acetylation							
Lysine (Kac)							
Writer	H3 (14,18,27), H4 (5,8), H2A (5), H2B (12,15)	Activation	CBP/p300	Human endogenous promoter	CRISPR/Cas9, ZF, TALE	p300 FL, CD	[53]
				Reporter plasmid in mammalian cells	Gal4	p300 FL, CD	[131]
				Integrated reporter in mammalian cells	LexA	p300 HAT	[132]
	H3 (9,14,18)	Activation, DNA repair	PCAF, GCN5				
	H4 (5,12)	Histone deposition	HAT1				
	H4 (5,8,12,16), H3(14)	Activation, DNA repair	TIP60				
	H4 (5,8,12)		HB01				
Eraser	H3 (9, 56), H4 (8, 16)	Repressive chromatin establishment, metabolism	SIRT1, SIRT2, SIRT3, SIRT5, SIRT6	Integrated reporter in human cells	Gal4	SIRT1 FL	[133]
Methylation							
Lysine (Kme)							
Writer	H3 (4)	Activation	MLL (1,2,3,4,5), SET1 (A,B), ASH1	Reporter gene in flies	Gal4	Ash1 FL, CD	[134]
	H3 (36)	Repression, transcriptional elongation	SET2, NSD1, SYMD2	Integrated reporter in yeast	LexA	Set2 FL, CD	[135]
	H3 (79)	Transcriptional elongation, euchromatin	DOT1				
	H3 (9)	Repression, imprinting	SUV39H (1,2), G9a, RIZ1, ESET/SETDB1, EuHMTase/GLP, CLL8, SETDB1	Human endogenous promoter	ZF	G9a CD, SUV39h1 FL, CD	[54]
				Integrated reporter in mammalian cells	Gal4	G9a FL	[136]
				Reporter plasmid in mammalian cells	Gal4	SUV39h1 FL, CD	[137]
	H3 (27)	Repression	EZH1/2, WHSC1				
	H4 (20)	Repression, activation, DNA repair, cell cycle	Pr-SET(7,8), SUV4 20H(1,2)				
Eraser	H3 (4)	Downregulation of proximal genes	LSD1, BHC110, jumonji class	Human endogenous enhancer	TALE	LSD1 FL	[32]
	H3 (36)		JHDM 1 (a, b), JMJD2A/JHDM3A, JMJD2C/GASC1				
	H3 (9)		JHDM 2 (a, b), JMJD2 (B, D), JMJD2A/ JHDM3A, JMJD2C/GASC1, LSD1				
Arginine (Rme)							
Writer	H3 (2, 17, 26)	Activation	CARM1				
	H4 (3)	Activation	PRMT4				
Ubiquitylation							
Lysine (Kub)							
Writer	H2B (123/120)	Activation	RNF (20, 40)				
	H2A (119)	Repression	Bmi/Ring1A				
Phosphorylation							
Threonine (Tph)							
Writer	H3 (3)	Mitosis					
Serine (Sph)							
Writer	H3 (28)	Immediate-early activation	MSK (1,2)				
	H4 (1)	Mitosis					
	H2A (139)	DNA repair	ATR, ATM, DNA-PK				
	H2B (14)	Apoptosis	Mst1				
Proline isomerization							
Proline (Pisom)							
Writer	H3 (30, 38)	Activation/repression	ScFPR4				

Activation, transcriptional activation; repression, transcriptional repression. See also [34] for examples of engineered histone deacetylases (HDACs), methyltransferases (HMTs), acetyltransferase (HAT) inhibitors, and HDAC- and HMT-recruiting proteins. Other useful references and guides [3, 4, 138, 139]. Abbreviations: *TALE* transcription activator-like effector, *ZF* zinc finger

Early examples of epigenome editors include programmable DNA methyltransferases [25–27] and demethylases [28–31], histone methyltransferases and demethylases [32–34], and histone acetyltransferases and deacetylases [33]. In addition, the use of transcriptional activators or repressors that have been reprogrammed to target specific loci can initiate chromatin-mediated changes. For example, ZF fusions to the Krüppel-associated box (KRAB) repressor domain of the transcription factor Kox1 have been shown to suppress the expression of endogenous target genes, such as *Sox2*, in breast cancer cells through chromatin modifications [35]. The KRAB domain recruits co-repressor KAP1 (KRAB-associated protein 1), which in turn assembles a repressive state by nucleosome-remodeling and deacetylation (NuRD), de-acetylation of histones, incorporation of H3K9me3 (SETDB1), and ultimately heterochromatin formation [36, 37]. Other approaches have used the chromoshadow domain of heterochromatin protein 1 (HP1) to induce heterochromatin formation when targeted to a defined locus by ZFs [38] or LacI [39]. Similarly, fusions to the p65 domain of the mammalian transcription factor NFkB have been used to activate a variety of endogenous genes (and transgenes), principally by promoting histone acetylation via recruitment of p300/CBP [40].

### Genomic specificity

Ideally, the activity of an engineered epigenome editor is localized to a specific genomic location. One key way of controlling this is through the DNA-targeting module. Indeed, the targeting specificity of the DNA-binding module is likely to be important in defining the overall activity of an editor, specifically by directing CR activity to a specific genomic locus and thereby minimizing opportunities for off-target effects. Studies that directly compare the activity of an editor across the different classes of DNA-binding modules are lacking, but different patterns of off-target activity have been detected, for example, for KRAB fusions to ZFs and nuclease-null dCas9 [41–43].

The genome-wide specificities of programmable DNA-binding modules, and strategies for improving them, have been the subject of considerable recent study [15, 44], which will not be discussed here. Epigenome editing will certainly benefit from these strategies, which include directed evolution [45], reducing non-specific DNA-binding energy [46, 47], truncating guide RNAs (gRNAs) in CRISPR systems [48], and structure-guided rational protein engineering [49, 50].

The genomic specificity of an editor can also, in some cases, be enhanced by altering the activity of the CR by changing its catalytic activity or its intrinsic interactions with binding partners, such as other regulatory proteins or DNA [41]. For example, for ZF fusions of DNA methyltransferases, mutants that had reduced catalytic activity gave rise to methylation that was more specific to targeted sites than that in the wild type [51, 52], presumably because the catalytic activity of the editors was more dependent on DNA binding.

### Biochemical specificity

The use of full-length CRs and potent transcriptional activators or repressors, such as KRAB and p65, can be effective in inducing chromatin-mediated transcriptional changes. However, these components are known to recruit multiple chromatin-modifying activities and to induce broad chromatin changes, which confound our ability to link specific modifications to specific functional roles. Addressing this issue requires epigenetic editors that have precise control over the desired chromatin-modifying activities. It also requires quantifying the biochemical specificity of an epigenetic editor, that is, quantifying the full array of modifications made to a locus that has been targeted by an editor. These modifications are inherently more challenging to quantify than genomic specificity: a comprehensive panel of DNA histone modifications must be assessed using techniques such as chromatin immunoprecipitation (ChIP) with many different antibodies.

Strategies to create epigenetic editors that have improved functional or biochemical specificity have been explored. One key strategy is to truncate chromatin-modifying enzymes to their catalytic core domains. A notable recent example involved the human co-activator protein p300, which functions as a histone acetyltransferase and mediates interactions with multiple transcription factors to regulate many genes in tissues throughout the body. By fusing the catalytic core of the p300 acetyltransferase to dCas9, Hilton et al. [53] created a programmable histone acetyltransferase. They showed that this minimal fusion protein was able to catalyze the acetylation of H3K27 at target promoter sites, which led to robust transcriptional activation of target genes. This elegant study provides strong support for histone acetylation as a causal mechanism for transcriptional activation, but it also highlights the challenges associated with functionally annotating specific chromatin modifications. In this particular study, it remained unclear whether H3K27 acetylation causes the observed transcriptional effects or whether another histone lysine at the site (or perhaps even a lysine residue on a completely different protein) causes these effects. These efforts would benefit from new and improved methods for quantifying biochemical specificity in the context of epigenome-editing experiments.

A related strategy for improving the functional specificity of epigenetic editors is to remove non-catalytic domains or components from CRs in order to minimize the potential for non-specific interactions. For example, site-specifically recruiting the minimal catalytic domain of the histone methyltransferase SUV39H1 with a ZF array efficiently repressed the VEGF-A promoter, whereas full-length SUV39H1 did not cause repression [54]. Presumably this was because the intact HP1 interaction domain present in full-length SUV39H1 functioned to titrate the protein away from the VEGF-A gene. Related examples include coupling of the catalytic domains of chromatin-modifying enzymes to dCas9 [53], ZFs [25, 40, 53–59], TALEs [33, 53, 60, 61], or use of the Gal4 DNA-binding domain [26] to repress or silence endogenous genes.

Collectively, these studies have used fusions to minimal catalytic domains to develop epigenetic editors that have improved functional specificity. Efforts to truly isolate and re-engineer the catalytic domains of CRs will be key to improving the functional specificity of epigenetic editors.

### Ongoing challenges

In addition to improving biochemical and site specificities, several important challenges remain. Current efforts have been focused predominantly on constructing epigenome editors by fusing writer or eraser domains with DNA-targeting elements. Engineered readers remain largely underdeveloped (Table 2). Potential applications of epigenomic readers include in vivo reporting on aberrant or disease-related modifications. An in vivo ChIP approach could feedback to an epigenome effector for reconfiguration of a detected aberrant modification state. In one example, a synthetic transcription factor was engineered by fusing the VP64 activation domain to the Polycomb chromodomain (PCD) [62]. The PCD of this synthetic transcription factor recognizes H3K27me3 that is associated with silenced genes and reactivates these genes. Engineering readers remains challenging for two reasons. First, it may be difficult to engineer a single histone reader domain that is specific for a particular histone residue. Combining multiple different reader domains, which is a common mode of natural chromatin regulation, may solve this problem. Second, as all similarly modified nucleosomes will look alike to chromatin readers, the readers will bind modifications throughout the genome rather than at specific locations. A combination of DNA- and chromatin-binding modalities may provide a solution. Given the complexity of chromatin biochemistry, there are probably many other features that will be important for the design of future epigenome-modifying tools. For example, histone lysine residues can exist in mono-, di-, and trimethylated states. Being able to finely tune this feature of chromatin modification could reveal its functional role and potentially provide fine-tuned control of transcriptional activity.

**Table 2:** Molecular readers of chromatin modifications

Reader domain scheme	Modifications	Example proteins
**Individual domains**		
Methylation		
Chromo	H3K4me2/3	CHD1
	H3K9me2/3	HP1, CDY1
	H3K27me3, H3K9me3	PC1/PC2/PC/LHP1
	H3K36me3	MSL3
Tudor	H3K4me3/H4K20me3	JMJD2A
	H4K20me2	PHF20
MBT	H3K4me1/ H4K20me1	PHF20L1
	H4K20me1/2, H3K20me, H3K9me	L3MBTL1
PHD	H3K4me3	TAF3
	H3K4me2/3	ING2, BPTF/dmNURF301
	H3K9me3	CHD4
Acetylation		
Bromodomain	H4K16ac	GCN5
	H3K23ac	TRIM24
	H3ac, H4ac, H4K16ac	P/CAF
Phosphorylation		
14-3-3	H3S10ph, H3S28p	14-3-3 ζ
**Multisite binding**		
Double tudor domain	H3K4me2, H3K9me2, H4K20me2	53BP1
Hybrid tudor domains (HTDs)	H3K4me2/3, H4K20me2/3	JMJD2A
Chromodomain Y-chromosome (CDY) [74]	H3K9me3, H3K27me3	
**Multivalent binding**		
Bromo + Bromo	H4K5ac + H4K12ac	TAF1 (subunit of TFIID)
PHD + Bromo	H3K4me3 + H4K16ac	BPTF
	H3K9ac/H3K12ac + H3K4me3	TFIID
Tudor + Bromo	H3K9ac/H3K14ac + H3K4me3	SAGA complex

Other useful references and guides [4, 140–142]

Continued work on characterizing and discovering new catalytic domains will expand the list of available parts from which to select for improved properties such as substrate specificity [63–71]. Another interesting approach for improving the catalytic activity of epigenome editors is to fuse the catalytic core domains of multiple subunits or co-recruiting synergistic co-factors. For example, fusion of the catalytic C-terminal domains of DNA methyltransferase 3a (DNMT3a) and DNMT3L induced DNA methylation at the VEGF-A promoter with better efficiency than did the DNMT3a catalytic domain alone, by mimicking a stable Dnmt3a–Dnmt3L heterodimer [59]. DNMT3L, despite its lack of catalytic activity, directly interacts with and stimulates the catalytic activity of DNMT3a. Targeting chromatin modification by coupling multiple subdomains that have catalytic or structural functions may be a better reflection of the natural mode of chromatin regulation.

## Combination and context

There exist a surprisingly large number of epigenome modifications. The combinatorial interactions between these modifications and other chromatin-bound proteins increase this complexity even further. In fact, most chromatin states that are associated with regions such as active promoters and enhancers are characterized by specific combinations of chromatin modifications [72]. Why did this combinatorial complexity evolve? One reason could be that single modifications alone are not sufficient to account for all the distinct states that need to be specified or marked. Perhaps a more intriguing possibility is that combinatorial interactions set the stage for context-dependent regulation and enhance locus-specific recruitment.

With context dependency, one modification could mask, modulate, or enhance the binding interaction of a reader of a second modification. This is seen in the association of HP1 with H3K9me3, which is abolished by the dynamic and transient phosphorylation of the adjacent Ser10 residue [73]. Similarly, association of the double chromodomains of CHD1 with H3K4me3 is reduced by demethylation of Arg2 (a two-fold reduction) or by phosphorylation of Thr3 (a 25-fold reduction). Trans-histone crosstalk can also occur, as found in COMPASS (Complex of Proteins Associated with Set1), the yeast homolog of the mammalian MLL complex [74]. A global functional proteomic screen revealed that monoubiquitination of histone H2B by Rad6 is required for H3K4 methylation by COMPASS and for H3K79 methylation by Dot1 [75]. The recruitment of Cps35, an essential subunit of COMPASS, to chromatin in the presence of H2B monoubiquitination facilitates the recruitment of COMPASS and Dot1. Thus, combinatorial modifications may act as gates, allowing events to occur only in a particular order.

Combinatorial modifications could also prime a gene to follow one of multiple possible paths. Certain domains of the embryonic stem (ES) cell genome possess both activating and repressive histone modifications, known as bivalent domains; these are typically enriched at developmentally important genes [76, 77]. It is proposed that genes that have bivalent domains are poised for either activation or repression, depending on the differentiation path that the cell ultimately follows.

Gene expression is precisely controlled in time and space by the integration of this diverse array of PTM signals and the actions of multiple chromatin-regulating factors operating in multifactorial ways [3, 78]. If we can design epigenome editors to control these complex states, we may be able to fully unveil the context dependency of chromatin regulation and thus understand whether the pre-established chromatin context will affect (cancel out, enhance, or synergize) the effectiveness of the following chromatin regulation. We might then be able to adopt the true combinatorial features of natural chromatin communication in a range of applications.

### Combinatorial and high-throughput techniques reveal contextual and combinatorial principles

The interactions between chromatin proteins, chromatin modifications, and the surrounding DNA sequence and chromatin state determine local transcriptional outputs. This is key for the design of functional epigenome editors because behaviors that are observed at one specific locus may not hold at another locus where the presence of existing proteins may alter the activity of a recruited epigenome editor. Therefore, one important goal for epigenome engineers is to reveal the rules of chromatin context. Accessing and deciphering these rules will require high-throughput and combinatorial techniques.

**Fig. 2:**
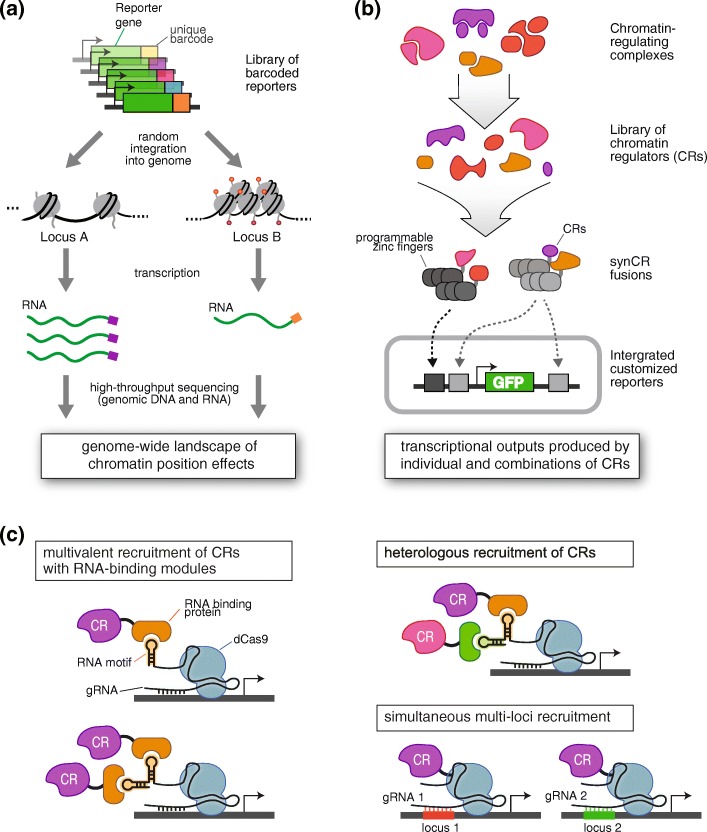
Interrogating the contextual and combinatorial principles of epigenome regulation. **a** A method for the parallel monitoring of the transcriptional activities of thousands of randomly integrated, barcoded reporters was used to study chromatin position effects across the genome. **b** Synthetic chromatin regulators (*synCRs*), composed of fusions of programmable zinc fingers (ZFs) and subunit proteins derived from diverse chromatin-regulating complexes, were used to study and program transcriptional outputs produced by individual and combinations of CRs at integrated reporters. *GFP* green fluorescent protein. **c** CRISPR/dCas9 can be exploited for high-throughput functional assays of epigenetic regulators thanks to its experimental tractability for combinatorial and multiplexed recruitment. Scaffolding multiple RNA-hairpin motifs to a guide RNA (*gRNA*) allows multivalent recruitment of chromatin regulators (*CRs*). Scaffolding different RNA motifs to gRNA allows heterologous recruitment of CRs. The same CR can be simultaneously recruited to multiple loci by using different gRNAs specific to each gRNA locus

There have been several in vitro methods for the rapid assessment of combinatorial and contextual properties of epigenome editors [79], but the intracellular and intranuclear environments are likely to have significant effects. To overcome the technical hurdles of working in the cellular environment, library-based methods can functionally assay comprehensive sets of regulators in vivo. For example, Akhtar and colleagues [80] randomly integrated thousands of barcoded reporter transgenes into the genome using piggyback transposition (Fig. 2a). By assaying cells with integrated reporters (IRs), these authors could test whether the local chromatin compaction state prior to integration had predictive power for IR expression levels. Analysis of normalized transgene expression by high-throughput sequencing of the library revealed non-random patterns of IR expression, which was strongly dependent on local chromatin context.

In our group, Keung et al. [81] fused a comprehensive set of 223 yeast CRs to programmable ZF proteins (Fig. 2b). We site-specifically co-recruited the CRs together with the commonly used transcriptional activator VP16 to diverse arrays of synthetic reporters. This revealed a range of transcriptional logic and behaviors, demonstrating the complexity of chromatin regulation. We divided this range of logic into six distinct classes of combinatorial regulation: dominant repressors, repressors, neutral factors, enhancers of VP16-mediated activation, additive activators, and synergistic activators.

### Future work

The simplicity of programming the CRISPR-Cas9 system to target multiple endogenous genomic loci simultaneously [82–84] and/or to recruit multiple different protein domains [85] to a locus offers a powerful platform with which to decipher the combinatorial and contextual complexity of the epigenome (Fig. 2c). The experimental tractability of CRISPR/Cas9 genome editing tools for high-throughput approaches exceeds that of any other currently available DNA-targeting platform [86–90]. Creatively leveraging previous systems could also expand the parameter space that is explored. For example, the platform that Akhtar and colleagues [80] developed could be adapted to study additional contextual effects. With only minor modifications in the experimental design, DNA sequence elements could be added or other chromatin modifiers recruited in front of the reporter gene to ask how each component interacts with each endogenous state.

## Memory and epigenetics

Among the myriad modifications being written and erased on chromatin, a subset is stably inherited through mitotic or meiotic cell divisions. These epigenetically inherited modifications are important for the maintenance of gene expression patterns throughout the differentiation and developmental processes of mammals and may result in disease or cancer when mis-regulated [8, 91]. Several important examples of behavioral and disease traits are inherited across generations in complex organisms, including mice [92]; here we focus on cellular studies because studies of the mechanistic roles of epigenome modifications are more feasible. Understanding and controlling epigenetic modifications could also have an impact on biotechnology and synthetic biology, where stable biological switches are highly desired.

A variety of different mechanisms underlie epigenetic properties but they all depend on some form of feedback. Broadly, feedback mechanisms can be *trans*- or *cis*-acting or a combination of both [93]. *Trans*-acting mechanisms typically involve positive feedback of a transcription factor in the regulation of its own gene. This mechanism is utilized both to establish and to self-sustain a specific transcriptional state of a gene, as demonstrated in the activation and maintenance of differentiated functions of nematode sensory neurons [94, 95] and widely in maintaining differentiated cell identity [96, 97]. *Cis*-acting mechanisms more often involve chromatin modifications directly. DNA methylation in mammals is a prime example [98]. DNA methylation is crucial in the establishment of epigenetic memory that is essential for normal development [99, 100]. Work in vertebrates has been focused mostly on the methylation of cytosine in the context of CpG di-nucleotides at transcription start sites (TSSs), which is believed to maintain genes in a locked-in off state. Recent advances in the genome-scale mapping of methylation has found additional context-dependent functions (in, for example, TSSs, gene bodies, and enhancers) that go beyond the repressive association of DNA methylation [101]. Epigenetic memory by DNA methylation is established through the DNA-strand to DNA-strand copying action of DNMT1 and through the recruitment of repressive regulatory proteins upon de novo methylation by DNMT3 [98]. However, this classic model for epigenetic memory, with the canonical distinction between the roles of DNMT3 and DNMT1, is being challenged by recent experimental evidence [102, 103].

Histone modifications are also involved in maintaining epigenetic regulation. For example, antagonizing groups of protein complexes, the Polycomb (PcG) and trithorax (trxG) groups, mediate the mitotic inheritance of repressive and active transcriptional states, respectively [104]. There is also evidence that some heterochromatic histone modifications crosstalk with and may derive their stability from DNA methylation [105, 106]. These examples point to the important role of chromatin in stably maintaining the transcriptional state of critical lineage-specifying genes. The exact mechanisms that underlie these epigenetic properties of chromatin modifications have been difficult to pin down given the time-dependent nature of gene-expression memory. Nevertheless, several temporally dynamic experimental approaches using epigenome editors have and will continue to shed light on the molecular feedback underlying memory in chromatin systems.

### Synthetic systems can directly induce epigenetic chromatin states

**Fig. 3:**
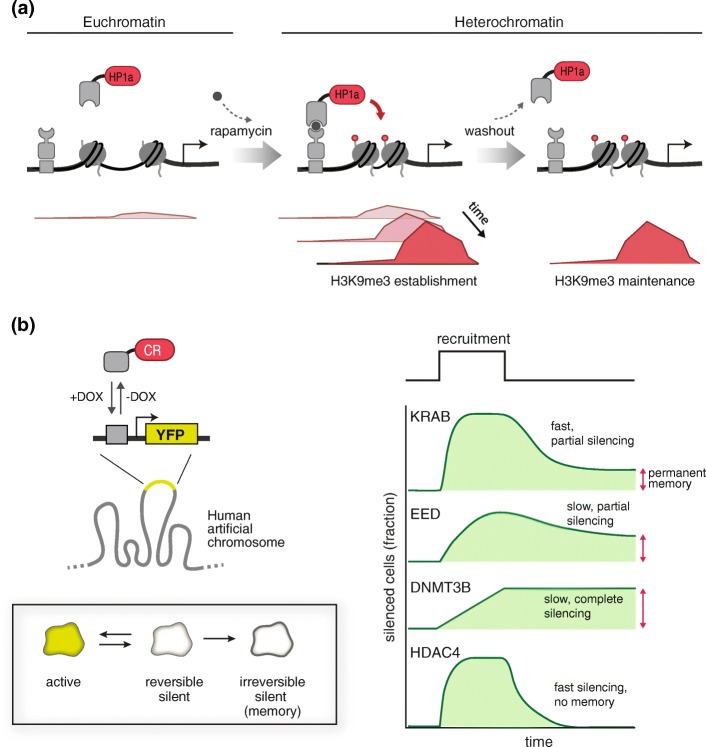
Use of epigenome editing tools to study the dynamics and memory of epigenetic regulation. **a** The selective recruitment of HP1α to specific loci in live cells was used to establish H3K9me3-dependent gene silencing and to study the kinetics and extent of heterochromatin. **b** In another study, doxycyline (*DOX*) was used to selectively recruit four repressive CRs that are associated with diverse chromatin modifications (Krüppel-associated box (*KRAB*) (associated with H3K9 methylation), embryonic ectoderm development (*EED*) (associated with H3K27 methylation), DNA methyltransferase 3B (*DNMT3B*) (associated with DNA methylation), and histone deacetylase 4 (*HDAC4*) (associated with histone deacetylation)). By tracking transcriptional output of a reporter gene in individual cells, researchers discovered that cells stochastically transition between active and silent states. These dynamics were described by a simple three-state model, in which different CRs operate over different time scales to modulate the fraction of cells in a population that are in each state. *YFP* yellow fluorescent protein

In a landmark study, Hathaway et al. [38] developed a chemically inducible system to establish and erase heterochromatin in vivo at the Oct4 locus (Fig. 3a). The chromoshadow domain of HP1α was site-specifically directed to ZFHD1-binding sites via FKBP-Frb dimerization domains in the presence of rapamycin. Upon transient recruitment of HP1α, a >10-kb region of H3K9 methylation was established and maintained through multiple cell divisions (over at least several weeks), even after the release of HP1α. By measuring the kinetics and stability of chromatin modification establishment and turnover, Hathaway et al. [38] generated a computational model that incorporated a feedback mechanism between DNA methylation and H3K9 methylation.

The relationship between DNA methylation and H3K9 methylation, as well as other types of repressive modifications, was further investigated by Bintu et al. [107] in an elegant synthetic biology study. These authors developed a framework to quantitatively interrogate the kinetics and stability of gene repression induced by four proteins that act through different types of chromatin modifications: (1) embryonic ectoderm development (EED) of Polycomb Repressive Complex 2 (PRC2) (H3K27 methylation), (2) KRAB (H3K9 methylation), (3) DNMT3B (DNA methylation), and (4) histone deacetylase 4 (HDAC4) (histone deacetylation) (Fig. 3b). Each protein was transiently recruited for different periods of time to a fluorescent reporter gene using the reverse Tet repressor (rTetR). Using single-cell time-lapse microscopy, Bintu et al. [107] observed that the reporter turned on and off in an all-or-none fashion for all of the chromatin modifiers studied. Yet the time it took for the reporter to turn off and the stability of repressed reporter differed depending on the modifier. In fact, each type of chromatin modification led to different kinetics and stabilities of gene repression, suggesting that the epigenome may encode different operational types of gene regulation.

The strong epigenetic properties of DNA methylation were confirmed in both studies. Nevertheless, studies are still attempting to confirm whether various histone modifications are truly epigenetic, that is, self-sustaining in the absence of the initial triggering signal or any necessary DNA sequence [95, 108, 109]. For example, the artificial recruitment of the PRC2 complex via a tetracycline-inducible GAL4–EED fusion protein induced H3K27me3, and this modification was maintained even after repression of GAL4–EED [110]. More recently, two studies have provided compelling evidence for the epigenetic inheritance of H3K9 methylation in the fission yeast *Schizosaccharomyces pombe* [111, 112]. A particularly important aspect of these findings was that the epigenetic inheritance of H3K9 methylation was decoupled from any DNA sequence and could be established at genomic loci that are normally devoid of H3K9 methylation and heterochromatin. In these two studies, the H3K9 methyltransferase Clr4 was recruited to the ade6^+^ gene [111, 112]. Transient recruitment of Clr4 was controlled by tetracycline-dependent release of TetR–Clr4. Interestingly, while the establishment of high levels of H3K9 methylation was subsequently lost upon release of the TetR-Clr4 initiator (within around ten cell divisions), deletion of the putative demethylase Epe1 resulted in H3K9-methylation-mediated silencing at the tethering site through many mitotic and meiotic divisions. These results suggest that the inheritance of H3K9 methylation is determined by the balance of a feedback loop between methylation by Clr4 through a reader–writer mechanism and active demethylation by Epe1. These studies demonstrate the synergy of forward-engineering approaches (such as those involving control of the genomic locus and of the timing of Clr4 recruitment) and chromatin biology techniques and genetics in demonstrating the factors required in the epigenetic maintenance of H3K9 methylation.

### Future work

Many other histone modifications still remain to be tested for their epigenetic properties and many molecular details of epigenetic mechanisms remain to be discovered [27]. These ongoing studies may benefit from technical advances that will make it possible to dynamically recruit proteins and to interrogate large parameter spaces in high-throughput screens for minimal factors that are required for epigenetic maintenance. For example, to identify the minimal factors required for epigenetic chromatin states, CRISPR-Cas9 systems could be used either to knockout chromatin proteins and/or to recruit multiple factors to specific genomic loci [38, 111–113]. In addition, greater temporal control could provide more precise information on the stability and kinetics of epigenetic systems. This could be achieved through the use of light-activated protein systems. Konermann et al. [33] demonstrated that 32 repressive histone effector domains could be conditionally targeted to a genomic locus via the light sensitive cryptochrome 2 (CRY2) protein and its interacting partner CIB1 from *Arabidopsis thaliana* [33]. This particular study was not focused on identifying the epigenetic properties of the chromatin modifiers, but this technique holds potential as a toolkit that can provide high temporal resolution with which to study epigenetic mechanisms and identify epigenetic factors [114].

Many opportunities for exploiting the unique features of epigenetic regulation lie ahead. Researchers could work to harness any potential restricted or conditional epigenetic inheritance of histone modifications for developing “short-term” or “flexible” epigenetic memory circuitry [99], which could be intentionally designed to maintain the edited epigenome state for a short period of time. For example, there may be instances, in normal development or for transient therapeutic applications, that require that genes are regulated such that they are suppressed for a short period of time and subsequently reactivated. The repressive state of a gene could be induced with repressive histone methyltransferases and later (before one cell cycle is completed or within very few cell divisions) reversed by either demethylases or a passive histone dilution mechanism. By contrast, complete and permanent repression of genes could be achieved with the incorporation of DNA-methylation-mediated gene silencing [25, 56]. It is important to note that there is evidence to suggest that transiently induced DNA methylation is not maintained, highlighting the importance of multivalent deposition of functionally related epigenetic marks for truly stable reprogramming [57]. Either short-term or long-term epigenetic memory could be a valuable feature of many applications, including gene and cell therapy. Finally, while the epigenetic maintenance of chromatin and gene expression states has been demonstrated in several cellular systems, exciting but challenging work lies ahead in using epigenome editing tools to study the long-term heritability of chromatin modifications (such as DNA methylation [92, 98]) across generations of complex organisms such as mice.

## Artificial perturbations of chromatin structure

Chromatin adds a unique spatial element to gene regulation at multiple scales [115, 116]. For example, certain histone modifications have been observed to demarcate and preserve chromatin domains such as silent heterochromatic and active euchromatic regions. These regions are hypothesized to be established and preserved by highly dynamic processes involving histone modifications; these include self-reinforcing mechanisms that spread modifications along adjacent nucleosomes [111, 112], so-called “reader-writer” mechanisms [117]. Chromatin’s three-dimensional conformation and positioning in the nucleus also orchestrate gene expression. For example, looping mediates long-range genomic interactions by juxtaposing distal regulatory elements such as enhancers with distant loci, to either coordinate their expression or co-localize regulatory factors. This type of spatial organization is observed in tissue-specific gene regulation, in which genomic elements cluster together at certain stages of development [118]. Tools that can replicate or perturb chromatin’s spatial properties will enhance our ability to study and potentially harness these complex mechanisms.

Several molecular approaches have already been used successfully to perturb chromatin structure and these studies suggest that continued work in this area could reveal important and potentially useful regulatory principles relating to chromatin shape. For example, an ectopic repressor assay using a drug-inducible ZF-KRAB fusion protein demonstrated that KRAB-mediated repression spans tens of kilobases and is established by the long-range propagation of H3K9me3 and HP1 β [119]. This and similar approaches [38, 81] provide us with the unique ability to regulate multiple genes in tandem using a single regulator. Furthermore, transcriptional activators and repressors that are recruited site-specifically to regions more than 1 kb downstream of promoters can activate [120] and repress [121] yeast genes, respectively, when they are placed near telomeres. This effect “at a distance” is mediated by a telomere-position effect in yeast, which is analogous to the position effect variegation (PEV) observed in *Drosophila*, wherein a normally active euchromatic gene is juxtaposed with heterochromatin by structural rearrangement and becomes silenced [122]. Modeling efforts along with site-specific recruitment approaches have also provided insights into how multiple regulators that have opposing functions (active or repressive) are coordinated to regulate genes in a way that is determined by the spatial distribution of nucleation sites along the chromosome [123, 124]. These studies can help to explain the expression pattern of adjacent genes in a certain positioning context and could potentially unveil the mechanisms of variegated gene expression.

Recent efforts have begun to directly manipulate chromatin looping and to change the three-dimensional contact profile of genes with other loci or nuclear structures (Fig. 1). Deng and colleagues [125, 126] employed ZFs to override a stringent developmental gene expression pattern by artificially forcing chromatin looping. Specifically, these researchers forced chromatin looping between the β-globin gene and its distal regulatory region, the locus control region (LCR) which is positioned 40 kb away. This looping was induced by synthetically tethering Ldb1, a protein present at the LCR, to the β-globin promoter, which led to Ldb1–Ldb1-mediated chromatin looping. Deng and colleagues demonstrated that forced chromatin looping was sufficient for activation of the β-globin gene [125, 126]. They then showed that forced chromatin looping that was achieved by tethering Ldb1 to a developmentally silenced embryonic globin gene was sufficient to trigger the gene’s reactivation. These studies demonstrate a novel approach to control the three-dimensional structure of the epigenome.

There are other ways to induce structural perturbations in chromatin. Even a change in the direction of a small fragment (~20 bp) of DNA sequence can control transcriptional activity by reconfiguring the topology of chromatin loops [127]. CCCTC-binding factor (CTCF) insulators and the associated cohesion complex play important roles in higher-order chromatin organization in mammalian genomes. By reversing the relative orientation of CTCF-binding sites by CRISPR/Cas9-based genome editing, changes in the directionality of DNA looping and gene expression can be made [127]. Such efforts will be key to elucidating the relationship between DNA sequence elements and the three-dimensional structure of chromatin.

Structural- or spatial-factor-dependent regulation of gene expression can also be mediated by spatially positioning genes in the nucleus. The randomly integrated reporter platform of Akhtar and colleagues [80], for example, revealed spatial positioning effects that correlated with gene expression. Lamina-associated domains (LADs), late-replicating domains, and regions marked by the histone modification H3K9me2 often coincide with one another and harbor mostly inactive endogenous genes [128]. In addition, the integrated reporters, much of whose fold change was unaccounted for by local chromatin compaction, were more actively expressed when integrated near active genes. Akhtar and colleagues proposed that these effects are a result of the collective actions of enhancers and transcription units in creating transcription-promoting regions, again highlighting the functional importance of how genes are spaced along a chromosome.

## Concluding remarks

In this review, we have discussed important features that must be considered when designing functional epigenome engineering tools and current challenges that need to be addressed. The impact of recent advances in epigenome engineering has been remarkable in terms of both understanding the underlying mechanisms of epigenome regulation and designing new ways to regulate genes for future biomedical and biotechnological applications. Forward-engineering approaches allow researchers to directly interrogate the relationships between the epigenome and transcriptional function. These approaches are highly complementary to other cell biology methods and are particularly useful for systematically exploring large parameter spaces [9]. In addition, epigenome editing technologies hold considerable promise for engineering applications. The application of engineering principles toward the construction of novel biological systems (i.e., synthetic biology) could take advantage of this additional class of chromatin-based regulation. The many features of epigenome regulation present interesting properties or functional connections that could be exploited in assembling synthetic biological networks [10]. Ultimately, epigenome editing may emerge in new forms of gene therapy by modifying/correcting diseased epigenome states without making permanent and potentially deleterious genetic changes in cells [8, 26, 129].

Perhaps one of the most exciting prospects in developing new epigenome editing tools is how they may alter our perspective of the function and nature of the epigenome’s complexity. Several current models depict chromatin modifications as an additional layer of regulatory nodes that act in concert with genetic networks to coordinate cellular programs [130]. With our increasing ability to interface, perturb, and establish these regulatory nodes, we can begin to think of the epigenome as a powerful set of operations that can be performed on signals from and between various levels of cellular regulation. Given the widespread use of the epigenome in nature, there is good reason to believe that epigenome editing, and the predictable manipulation of chromatin modifications, will serve as a powerful new paradigm for synthetic biology and bioengineering. No longer will the epigenome be a complex problem to decipher, but rather a powerful platform to harness.

## Abbreviations

ChIP: Chromatin immunoprecipitation
COMPASS: Complex of Proteins Associated with Set1
CR: Chromatin regulator
CTCF: CCCTC-binding factor
DNMT3a: DNA methyltransferase 3a
EED: Ectoderm development
gRNA: Guide RNA
HDAC4: Histone deacetylase 4
HP1: Heterochromatin protein 1
IR: Integrated reporter
KRAB: Krüppel-associated box
LCR: Locus control region
PCD: Polycomb chromodomain
PRC2: Polycomb Repressive Complex 2
PTM: Post-translational modification
TALE: Transcription activator-like effector
TSS: Transcription start site
ZF: Zinc finger
